# Structure-Guided Design of Novel Diarylpyrimidine-Based NNRTIs Through a Comprehensive In Silico Approach: 3D-QSAR, ADMET Evaluation, Molecular Docking, and Molecular Dynamics

**DOI:** 10.3390/ph18121854

**Published:** 2025-12-05

**Authors:** Mouna Baassi, Mohamed Moussaoui, Sanchaita Rajkhowa, Hatim Soufi, Rachid Daoud, Said Belaaouad

**Affiliations:** 1 Laboratory of Physical Chemistry of Applied Materials (LCPMA), Faculty of Sciences Ben M’Sick, Hassan II University of Casablanca, Casablanca 20670, Morocco; moussaouimohamed143@gmail.com (M.M.); hatimsoufi3@gmail.com (H.S.); sbelaaouad@yahoo.fr (S.B.); 2 Chemical and Biochemical Sciences-Green Processing Engineering, Mohammed VI Polytechnic University, Ben Guerir 43150, Morocco; 3 Centre of Biotechnology and Bioinformatics, Dibrugarh University, Dibrugarh 786004, Assam, India; s_rajkhowa@dibru.ac.in

**Keywords:** HIV-1, NNRTIs, 3D-QSAR, molecular docking, molecular dynamics simulation, ADMET, drug-likeness, SASA, MoLSA

## Abstract

**Background/Objectives:** The emergence of drug-resistant HIV-1 strains challenges the long-term efficacy of current antiretroviral therapies. Non-nucleoside reverse transcriptase inhibitors (NNRTIs) are critical in HIV-1 treatment; however, the need for new candidates with improved resistance profiles and pharmacokinetics remains. This study aims to design and evaluate novel NNRTIs targeting both wild-type (WT) and mutant-type (MT) HIV-1 reverse transcriptase (RT) using integrated computational strategies. **Methods:** We conducted a 3D-QSAR study on 33 naphthyl-diarylpyrimidine derivatives using CoMFA and CoMSIA models. We designed thirty-five novel molecules based on contour map insights. We applied ADMET and drug-likeness filters to prioritize ten candidates. Molecular docking was performed on WT (PDB: 3HVT) and MT (PDB: 4PUO) RT structures. The top candidates underwent 100 ns molecular dynamics (MD) simulations. We analyzed structural stability via RMSD, RMSF, and Rg, while we used SASA and MolSA to assess solvent exposure and surface compactness. **Results:** The CoMFA and CoMSIA models demonstrated robust predictivity (R^2^ = 0.979/0.920, Q^2^ = 0.643/0.546, R^2^_test_ = 0.747/0.603). P14 and P43 showed higher binding affinities than nevirapine and favorable ADMET profiles. MD simulations confirmed stable binding in WT-RT and adaptive flexibility in MT-RT. SASA and MolSA analysis revealed favorable conformational compaction. Drug-likeness profiles indicated optimal log P, strong hydrogen bonding, and acceptable bioavailability. **Conclusions:** P14 and P43 demonstrate strong potential as NNRTI leads, combining binding affinity, structural stability, and favorable pharmacokinetics, supporting further experimental development.

## 1. Introduction

AIDS stands for Acquired Immunodeficiency Syndrome. It is a severe state of weakened immune function as a result of the infection with the Human Immunodeficiency Virus (HIV), leading to the loss of over 35 million lives and currently affecting 38 million individuals worldwide [1].

The clinical treatment of HIV infection crucially involves a regimen of highly active antiretroviral therapy (HAART), which combines three or more types of antiretroviral drugs. This approach has demonstrated effectiveness in decreasing both the morbidity and mortality associated with HIV-1 infection [2,3].

HIV-1 reverse transcriptase (RT) emerged as a promising target for antiretroviral therapy early on because it is essential for the life cycle of HIV. It converts single-stranded genomic RNA into double-stranded DNA, which is subsequently integrated into the host chromosome and passed on to all progeny cells [4].

Therefore, the highly active antiretroviral therapy regimen, which typically involves well-known reverse transcriptase inhibitors, has demonstrated exceptional efficacy and safety in suppressing viral replication and mitigating the spread of the virus [5].

Currently, there are two approved classes of drugs designed to target HIV-1 reverse transcriptase (RT) for the treatment of HIV infection, which are nucleoside/nucleotide RT inhibitors (NRTIs and NtRTIs, respectively) and non-nucleoside RT inhibitors (NNRTIs). Both classes play vital roles in HAART.

In the current study, our focus will be on NNRTIs. They exhibit significant potential as effective inhibitors of HIV-1 reverse transcriptase [6].

However, flexibility in conformation and adaptability compromise their efficacy [7].

The FDA has approved six NNRTIs, including the first-generation nevirapine, efavirenz, and delavirdine, alongside the second-generation rilpivirine, etravirine, and dolutegravir [8].

Nevertheless, many drugs among them are not recommended according to current guidelines due to considerations related to their toxicity or antiviral effects as well as less effectiveness against several commonly observed mutant viruses, such as Y181C, Y188C, K103N, and L100I [9].

Therefore, there is an urgent need to develop and design new and more efficient NNRTIs with improved drug resistance profiles [7].

## 2. Results

### 2.1. Molecular Alignment

The molecular structures of the 33 selected compounds were generated and subjected to energy minimization using SYBYL-X 2.0 software [10]. The optimization was performed with the Tripos standard force field using a convergence criterion of 0.01 kcal/(Å·mol), and Gasteiger–Hückel atomic partial charges were applied [11].

**Figure 1:**
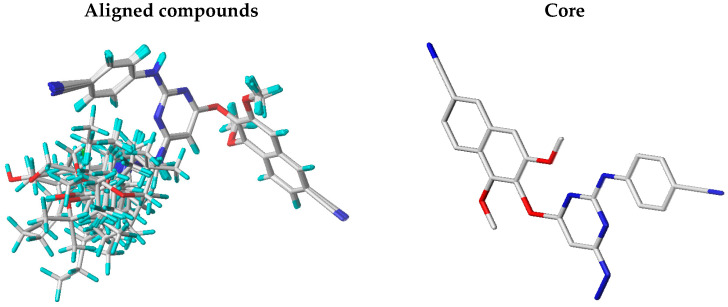
Superimposition of aligned compounds and identification of the common core using compound **12** as a template.

Following minimization, compound **12**, the most biologically active, was selected as the template for alignment. The remaining compounds were aligned to this reference structure using the alignment method available in SYBYL-X 2.0 to maintain a consistent molecular orientation across the dataset. The minimized and aligned structures, shown in Figure 1, were used as input for the subsequent construction of the 3D-QSAR models.

### 2.2. 3D-QSAR Models

CoMFA and CoMSIA strategies were applied to investigate the relationship between the 3D structures and biological activity of the compounds. The partial least squares (PLS) analysis results are presented in Table 1.

The CoMFA model achieved an R^2^ of 0.979 and a cross-validation Q^2^ of 0.643 for the training set. The model was constructed with five components (N = five), a standard error of estimate (SEE) of 0.067, and a Fisher value of 200.37. External validation using the test set provided an R^2^_test_ of 0.747. The contributions of the steric and electrostatic fields were 63.7% and 36.3%, respectively.

For the CoMSIA model, the training set produced an R^2^ of 0.92 and Q^2^ of 0.546, with four components (N = four), an SEE of 0.126, and a Fisher value of 66.51. The field contributions were 47% steric, 30.4% electrostatic, and 22.6% donor.

Table 2 presents the observed and predicted pIC_50_ values for both models. Residual values, defined as the differences between observed and predicted activities, were predominantly within ±0.2.

### 2.3. COMSIA and COMFA Contour Map Analysis

We employed compound **12**, the most biologically active molecule, as a reference to generate CoMFA and CoMSIA contour maps. These maps helped identify spatial regions where steric, electrostatic, and hydrogen bond donor interactions influence biological activity.

#### 2.3.1. CoMFA Contour Maps

**Figure 2:**
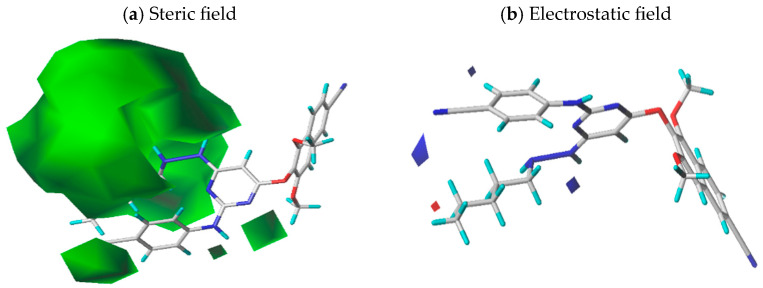
CoMFA contour maps based on molecule **12**: (**a**) steric field and (green) (**b**) electrostatic field (blue).

Steric and electrostatic field contributions are 63.7% and 36.3%, respectively (Figure 2). The steric contour map shown in Figure 2a highlights large green contours (80% contribution), indicating areas where bulky substituents enhance activity. The electrostatic map displayed in Figure 2b shows small blue contours (80% contribution) where electronegative groups favor activity, and a small red contour (20% contribution) indicating a region where electronegative groups reduce activity.

#### 2.3.2. CoMSIA Contour Maps

**Figure 3:**
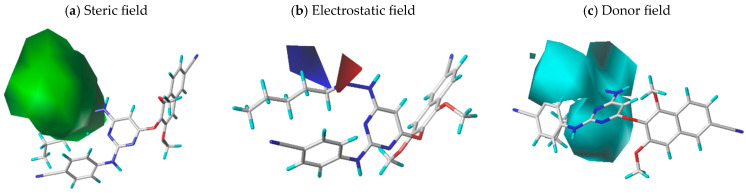
CoMSIA contour maps: (**a**) steric field (green), (**b**) electrostatic field (blue), (**c**) hydrogen bond donor (cyan).

In CoMSIA, the steric, electrostatic, and hydrogen bond donor fields contribute with 47%, 30.4%, and 22.6%, respectively (Figure 3). The steric contour map shown in Figure 3a reveals a large green contour (80% contribution), while the electrostatic map displayed in Figure 3b include small blue (80%) and red (20%) contours in adjacent regions. The donor contour map in Figure 3c displays three large cyan contours (80%), marking zones where donor groups enhance inhibitory activity.

### 2.4. Design of New Naphthyl-Diarylpyrimidines Derivatives

**Figure 4:**
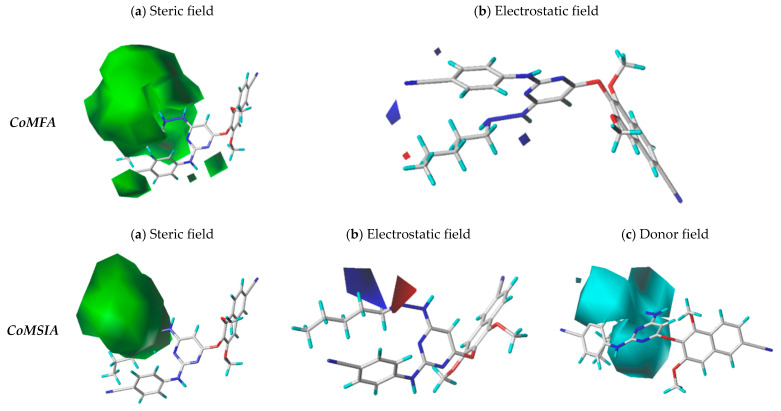
Grouped CoMFA and CoMSIA contour maps showing structural determinants for inhibitory activity.

**Figure 5:**
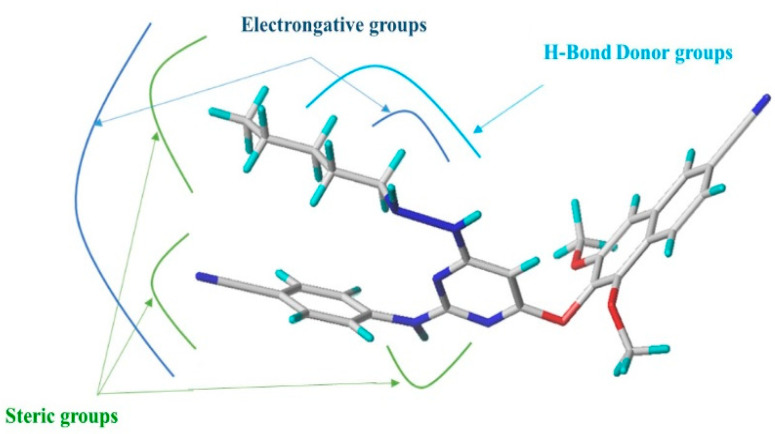
Structural interpretation of ligand features using CoMFA and CoMSIA contour maps: steric, electrostatic, and H-bond donor field contributions.

Based on insights from CoMFA and CoMSIA contour maps as illustrated in Figure 4, 35 novel derivatives of compound **12** were designed by targeting regions favorable for steric, electrostatic, and donor field interactions, as represented in Figure 5. We optimized and aligned these molecules using compound **12** as a structural template.

Table 3 presents the substituent patterns (R1–R8) of the newly designed NNRTI analogs and their predicted pIC_50_ values derived from CoMFA and CoMSIA models. We introduced the structural modifications based on contour map interpretations to enhance steric accommodation, optimize electrostatic interactions, and promote favorable hydrogen bond donor properties. Specific fragments such as t-butyl (C(CH_3_)_3_), hydroxyl (OH), amino (NH_2_), carboxyl (COOH), and carbonyl derivatives (COR) were incorporated at various positions to improve binding affinity and predicted activity.

The predicted pIC_50_ values for the new compounds, presented in Table 3, were higher than those of the reference compound **12**, indicating improved inhibitory potential against reverse transcriptase (RT).

### 2.5. ADMET Prediction

We evaluated the pharmacokinetic profiles of the ten newly designed molecules using in silico ADMET prediction models, covering absorption, distribution, metabolism, excretion, and toxicity. We summarized the results in Table 4.

#### 2.5.1. Absorption Parameters

We evaluated the absorption potential of the designed molecules based on two key indicators: water solubility and predicted intestinal absorption.

Based on the results shown in Table 4, all designed molecules exhibited water solubility values ranging from −2.89 to −3.22 Log mol/L, indicating moderate solubility in an aqueous solution [12,13,14].

The predicted human intestinal absorption values were above the 30% threshold for all compounds, except for P121, which presented lower absorption potential [15,16].

This suggests that the majority of the designed compounds possess high absorption potential in the human intestine, contributing to their overall bioavailability and therapeutic viability [17,18].

#### 2.5.2. Distribution Parameters

We evaluated the distribution potential of the designed compounds through central nervous system (CNS) permeability, using the blood–brain permeability surface area product (log PS) as the primary metric.

All compounds have proven log PS values within the optimal range of −5 to −4, as shown in Table 4, indicating adequate CNS penetration, except for compound P124, which fell outside this threshold [19]. These values suggest that the majority of the compounds are capable of effectively crossing the blood–brain barrier and reaching the central nervous system. These compounds’ ability to penetrate the CNS enhances their therapeutic effectiveness in such cases [20].

#### 2.5.3. Enzymatic Metabolism

We analyzed the metabolic behavior of the designed compounds with respect to their interaction with cytochrome P450 (CYP450) isoforms. The results in Table 4 show that compounds P14, P43, P120, and P124 are predicted to act as substrates for CYP3A4. None of the compounds exhibited inhibitory activity against the major CYP isoforms evaluated (1A2, 2C19, 2C9, 2D6, and 3A4) [21,22,23,24].

#### 2.5.4. Excretion and Total Clearance

As presented in Table 4, all ten designed compounds have proven total clearance values below 0.5 mL/min/kg, indicating a reduced rate of elimination and prolonged systemic persistence [16].

#### 2.5.5. Toxicity Assessment

The toxicological evaluation using AMES and hepatotoxicity predictive models indicated that all ten newly designed molecules are non-toxic; the results are shown in Table 4.

### 2.6. Drug-likeness Evaluation

We evaluated key physicochemical parameters associated with oral bioavailability and early-stage lead suitability. These parameters include the molecular weight (MW), lipophilicity (log P), number of rotatable bonds, hydrogen bond acceptors (HBAs), hydrogen bond donors (HBDs), and bioavailability score. The results for all ten designed compounds (P14 to P148) are highlighted in Table 5.

All compounds presented bioavailability scores of 0.17, with molecular weights ranging between 571.58 and 780.91 g/mol. Log P values were within the range of 1.53 to 4.07, indicating acceptable lipophilicity for membrane permeability. The number of rotatable bonds varied between 13 and 17, suggesting substantial structural flexibility. HBA values ranged from 11 to 15, while HBD values were between 6 and 9 across all candidates.

### 2.7. Molecular Docking Study

Following the ADMET evaluation, we excluded compounds P121 and P124 due to unfavorable pharmacokinetic properties. Molecular docking was subsequently performed on the remaining eight compounds using wild-type (WT) RT (PDB ID: 3HVT) and mutant-type (MT) RT (PDB ID: 4PUO). We compared the binding affinities of these compounds to that of the FDA-approved NNRTI, nevirapine (NVP). Among the tested molecules, P14 and P43 exhibited the highest binding affinities, surpassing those of NVP in both WT and MT forms. According to Table 6, P14 showed docking scores of −12.5 kcal/mol (WT) and −10.5 kcal/mol (MT), while P43 exhibited values of −13.0 kcal/mol (WT) and −10.7 kcal/mol (MT), compared to NVP’s −9.1 kcal/mol (WT) and −10.0 kcal/mol (MT).

**Figure 6:**
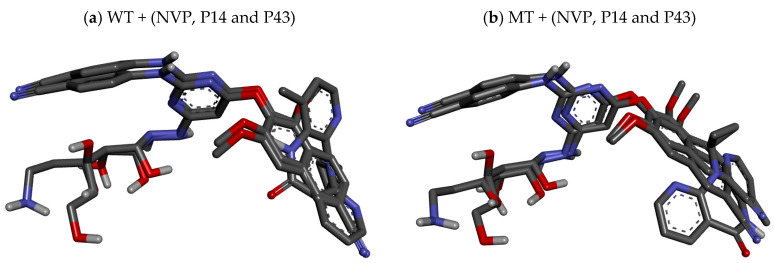
Superimposition of co-crystallized ligand nevirapine (NVP) and re-docked ligands (P14 and P43) in the active site of wild-type and mutant-type reverse transcriptase.

**Figure 7:**
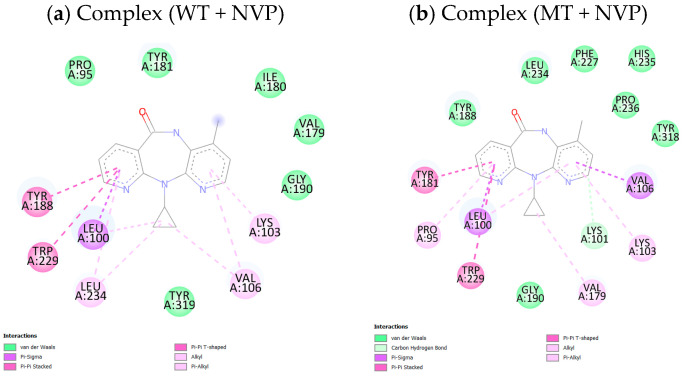
2D representation of ligand NVP interactions with wild-type and mutant reverse transcriptase receptors.

**Figure 8:**
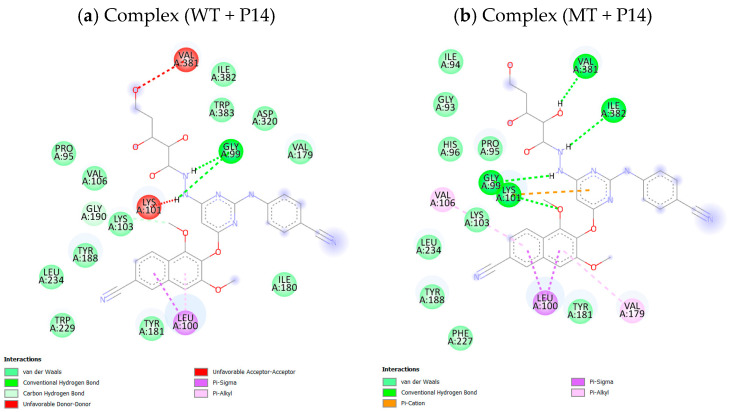
2D Representation of ligand P14 interactions with wild-type and mutant reverse transcriptase receptors.

**Figure 9:**
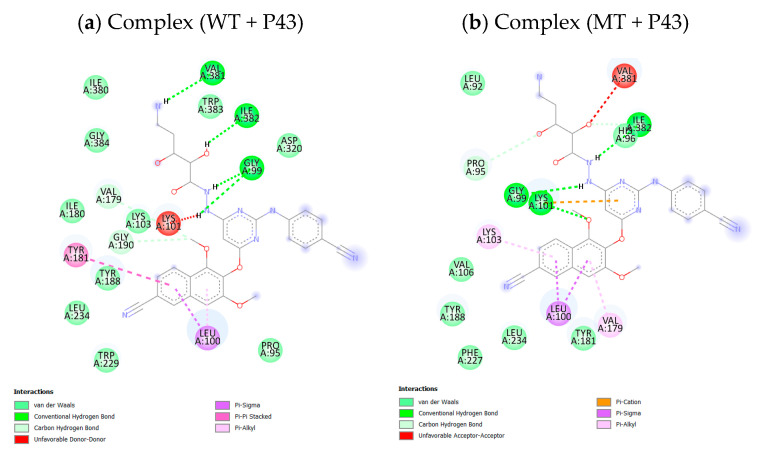
2D representation of ligand P43 interactions with wild-type and mutant reverse transcriptase receptors.

As displayed in Table 7, the SphereObject coordinate analysis showed that P14 and P43 adopted spatial positions within the active sites of WT and MT RT proteins, similar to those of the co-crystallized ligand NVP. Figure 6 represents the superimposition of docking poses, confirming a strong alignment between the native and re-docked ligands for both receptor types. The interaction assessment revealed that NVP primarily engaged in hydrophobic and Pi–Pi interactions, while P14 and P43 formed multiple conventional hydrogen bonds and Pi–cation electrostatic interactions, particularly with the LYS101 residue in the MT-RT variant (Figure 7, Figure 8 and Figure 9). Table 8 summarizes the active site residues and interaction types for each ligand–receptor complex, with P14 and P43 forming more stabilizing interactions than NVP.

### 2.8. Molecular Dynamics Simulation Analysis

#### 2.8.1. Wild-Type Native Protein

To assess the dynamic behavior and structural stability of the wild-type HIV-1 reverse transcriptase (WT-RT) protein and its ligand-bound complexes (WT + NVP, WT + P14, and WT + P43), molecular dynamics (MD) simulations were performed over a 100 ns timescale. The analysis focused on three key structural parameters: root mean square deviation (RMSD), root mean square fluctuation (RMSF), and radius of gyration (Rg) [25].

**Figure 10:**
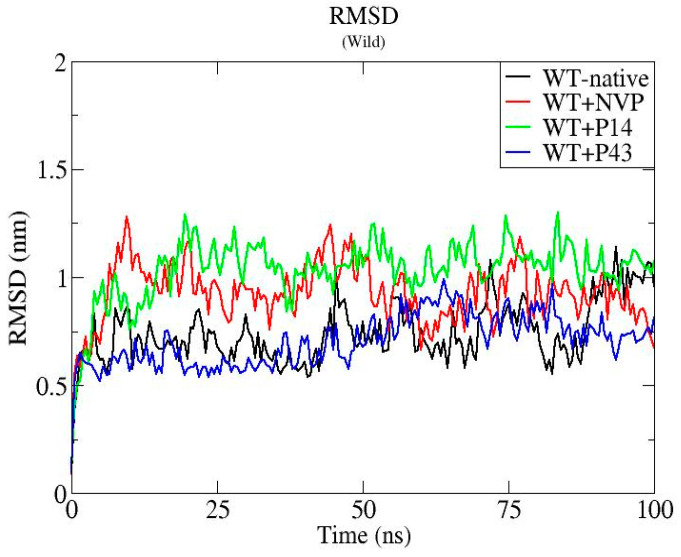
Root mean square deviation (RMSD) profiles of native wild-type reverse transcriptase and its complexes with nevirapine (NVP), P14, and P43 over a 100 ns of molecular dynamics simulation.

The RMSD analysis presented in Figure 10 indicates that the WT-native complex maintained structural stability throughout the simulation, with values ranging from approximately 0.5 to 1.1 nm. Binding with NVP and P43 resulted in similar or slightly improved stability, with WT + P43 exhibiting the lowest RMSD profile (~0.5 to 0.1 nm) and WT + NVP ranging between ~0.6 and 1.2 nm. In contrast, the WT + P14 complex showed the highest RMSD values (~0.6 to 1.4 nm), suggesting increased conformational deviation.

**Figure 11:**
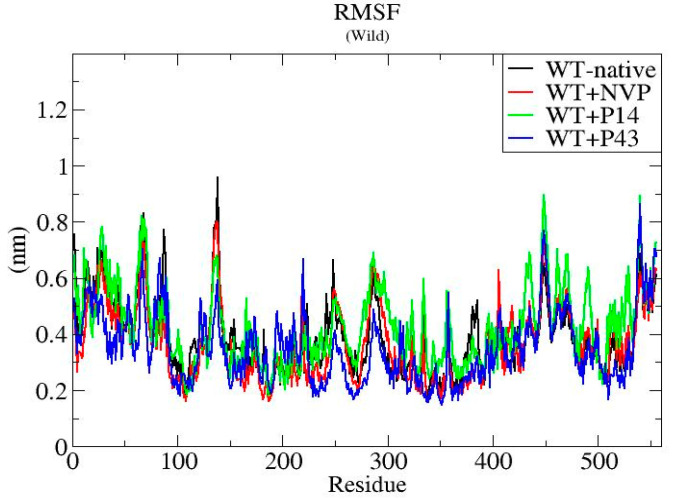
RMSF plots of native wild-type reverse transcriptase and ligand-bound complexes (NVP, P14, and P43) throughout the simulation period.

RMSF profiles displayed in Figure 11 for all complexes show comparable fluctuation patterns, with most residues fluctuating between ~0.2 and 0.8 nm. However, the WT + P14 complex demonstrated slightly higher flexibility in specific loop regions, which may contribute to its elevated RMSD profile.

**Figure 12:**
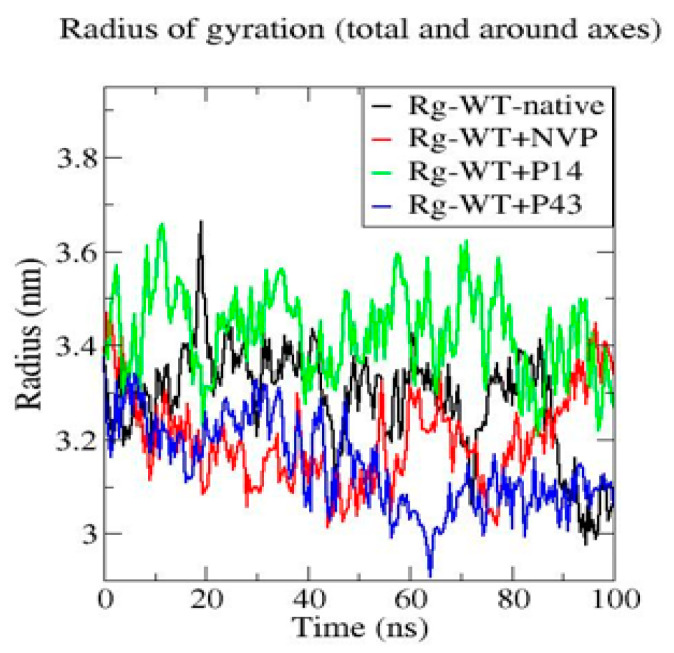
Radius of gyration (Rg) profiles of native wild-type reverse transcriptase and its complexes with NVP, P14, and P43 during molecular dynamics simulation.

The Rg analysis highlighted in Figure 12 reveals that the WT-native protein maintained compactness with values around ~3.2 to 3.4 nm. The WT + P43 complex showed the most compact profile (~3.0 to 3.3 nm), followed by WT + NVP (~3.1 to 3.4 nm). The WT + P14 complex exhibited the highest Rg values (~3.3 to 3.7 nm), indicating reduced compactness and a more expanded structural conformation.

We provide a summary of these parameters in Table 9, highlighting the comparative dynamic stability of each system.

#### 2.8.2. Mutant-Type Native Protein

**Figure 13:**
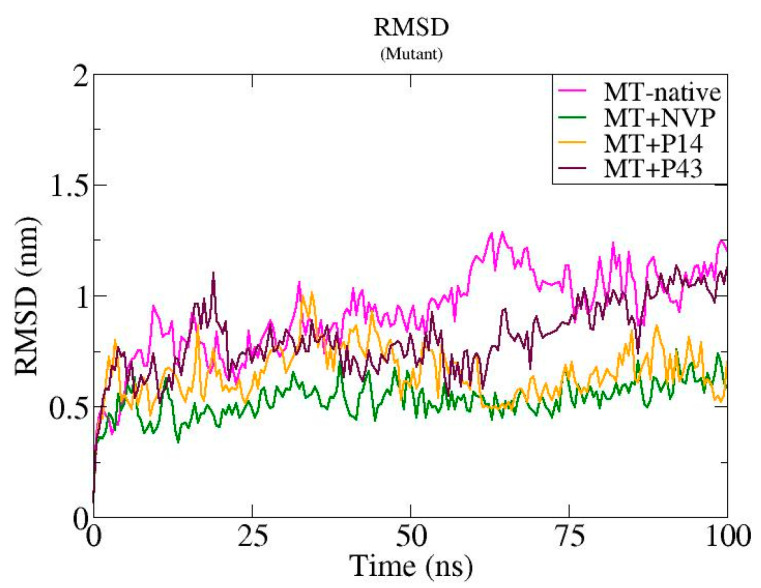
Root mean square deviation (RMSD) profiles of native mutant-type reverse transcriptase and its complexes with nevirapine (NVP), P14, and P43 over a 100 ns of molecular dynamics simulation.

**Figure 14:**
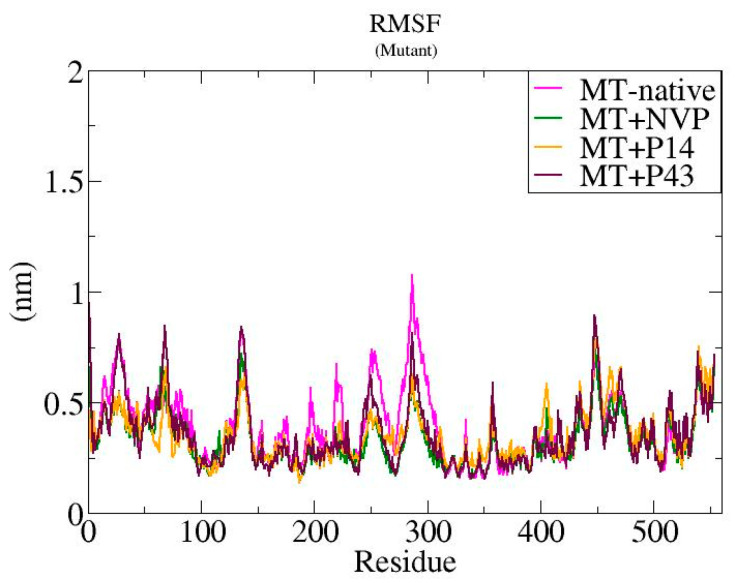
RMSF plots of native mutant-type reverse transcriptase and ligand-bound complexes (NVP, P14, and P43) throughout the simulation period.

**Figure 15:**
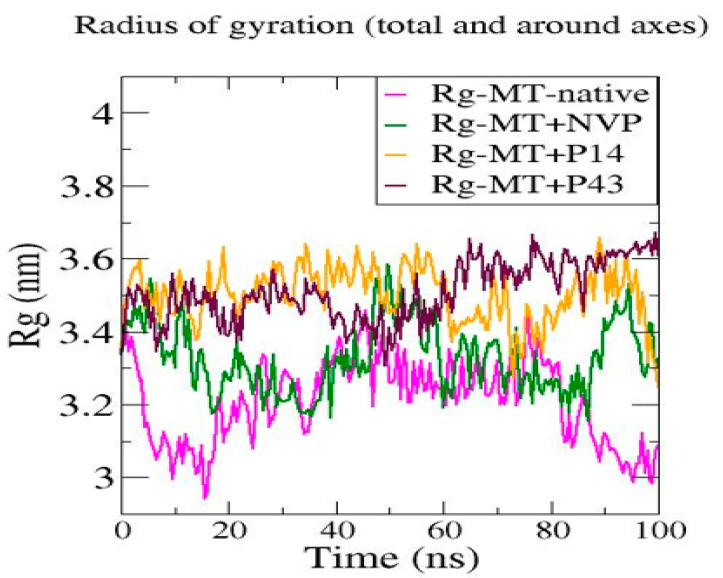
Radius of gyration (Rg) profiles of native mutant-type reverse transcriptase and its complexes with NVP, P14, and P43 during molecular dynamics simulation.

We performed molecular dynamics simulations over 100 ns to assess the structural stability of mutant-type reverse transcriptase (MT-RT) and its complexes with NVP, P14, and P43 [25]. As depicted in Figure 13, the root mean square deviation (RMSD) analysis indicated that the MT-native system exhibited the highest fluctuation, ranging between ~0.5 and 1.5 nm, while the MT + NVP complex showed the lowest RMSD values (~0.4–0.7 nm), suggesting improved structural stability. The MT + P14 complex displayed intermediate stability (~0.5–1.0 nm), and the MT + P43 complex showed fluctuations slightly lower than the MT-native complex but higher than those of MT + NVP and MT + P14. Figure 14 represents the root mean square fluctuation (RMSF) analysis revealed that the MT-native complex had the highest residue mobility, with peaks up to ~1.2 nm. In contrast, MT + NVP showed the lowest RMSF values, while MT + P14 and MT + P43 exhibited moderate flexibility reductions. The radius of gyration (Rg) values displayed in Figure 15 further highlighted these differences, with the MT-native complex maintaining relatively compact values (~3.2–3.3 nm), MT + NVP maintaining similar compaction (~3.1–3.3 nm), and MT + P14 and MT + P43 exhibiting expanded conformations (~3.4–3.6 nm and ~3.5–3.7 nm, respectively).

These findings are summarized in Table 10 and support the conclusion that nevirapine confers the greatest conformational stability to the mutant RT enzyme, whereas P14 and P43 induce moderate expansion and flexibility while maintaining acceptable structural integrity.

### 2.9. Surface Area Analysis: SASA and MolSA

We computed the solvent accessible surface area (SASA) and molecular surface area (MolSA) to investigate the structural behavior and surface exposure of the RT–ligand complexes during the 100 ns MD simulations.

#### 2.9.1. Wild-Type Native Protein

**Figure 16:**
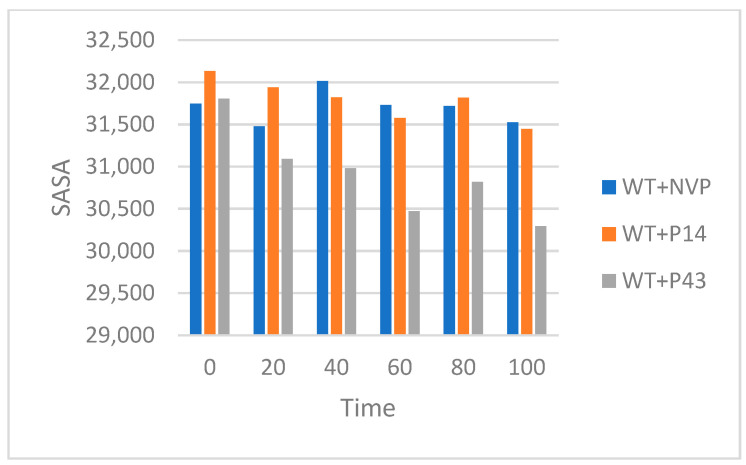
Solvent accessible surface area (SASA) of WT-RT complexes over a 100 ns MD simulation.

To evaluate the solvent exposure and molecular compactness of wild-type HIV-1 reverse transcriptase (RT) complexes, we analyzed the solvent accessible surface area (SASA) and molecular surface area (MolSA) over 100 ns molecular dynamics simulations, using snapshots extracted every 20 ns. The SASA values, which reflect the degree of surface exposure to the solvent, are summarized in Table 11. As demonstrated in Figure 16, the WT + NVP complex exhibited relatively stable SASA values throughout the simulation, ranging from approximately 31,500 Å^2^ to 32,000 Å^2^, indicating sustained conformational integrity and solvent accessibility. The WT + P14 complex showed a slightly higher initial SASA (~32,200 Å^2^), with values fluctuating between ~31,400 and 31,900 Å^2^ across the trajectory, suggesting moderate flexibility without significant destabilization. In contrast, the WT + P43 complex displayed a continuous decrease in the SASA from ~31,800 Å^2^ to ~30,300 Å^2^, reflecting a gradual reduction in the solvent-exposed surface area, which may be attributed to structural compaction or ligand-induced closure.

**Figure 17:**
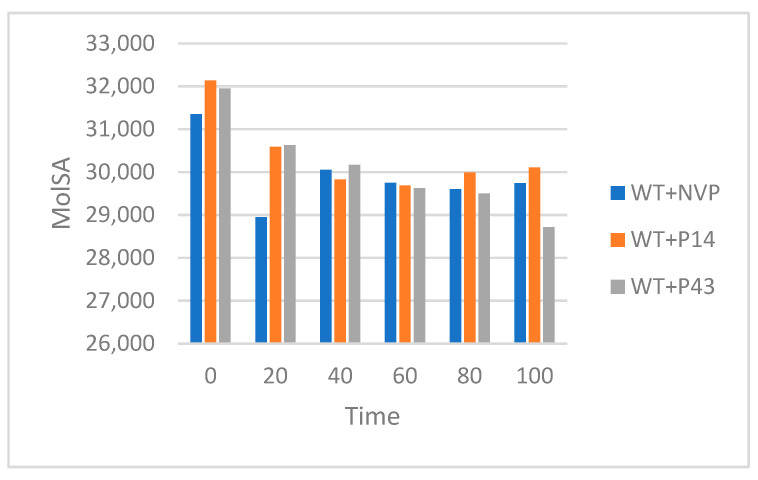
Molecular surface area (MolSA) of WT-RT complexes over a 100 ns MD simulation.

The MolSA values, which represent the molecular surface area and reflect structural compactness, are presented in Table 12. As depicted in Figure 17, the WT + NVP complex showed a gradual reduction in MolSA from ~31,300 Å^2^ to ~29,700 Å^2^, stabilizing after 40 ns, indicating progressive packing. A similar trend was observed for the WT + P14 complex, which declined from ~32,100 Å^2^ to ~30,100 Å^2^, highlighting a consistent and stable conformational arrangement. The WT +P43 complex experienced the most notable MolSA decrease, from ~31,900 Å^2^ at 0 ns to ~28,800 Å^2^ at 100 ns, suggesting enhanced compactness and a possible ligand-induced tightening of the protein’s structure.

#### 2.9.2. Mutant-Type Native Protein

We extended our study to analyze the solvent accessible surface area (SASA) and molecular surface area (MolSA) for the mutant-type HIV-1 reverse transcriptase (MT-RT) in complex with nevirapine (MT + NVP) and the proposed ligands P14 (MT + P14) and P43 (MT + P43) over a 100 ns simulation period.

**Figure 18:**
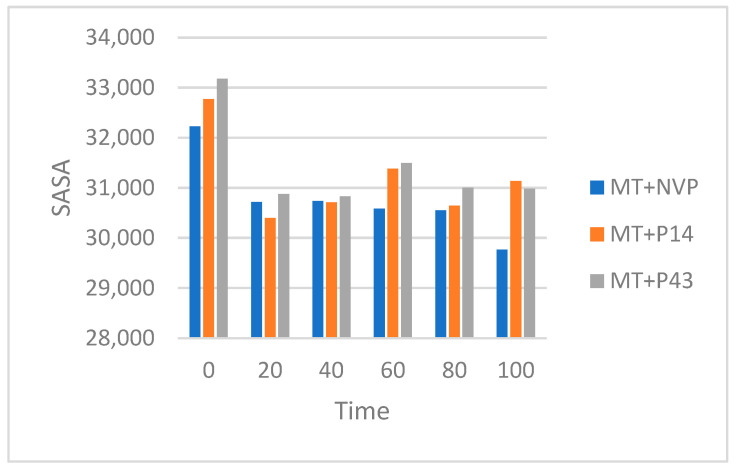
Solvent accessible surface area (SASA) of MT-RT complexes over a 100 ns MD simulation.

The SASA results, summarized in Table 13 and illustrated in Figure 18, show that the MT + NVP complex exhibits a gradual reduction in surface exposure, decreasing from ~32,200 Å^2^ at 0 ns to ~29,800 Å^2^ at 100 ns, indicating a moderate contraction and structural stabilization. MT + P14 shows an initial drop from ~32,800 Å^2^ to ~30,200 Å^2^ at 20 ns, followed by relatively stable values fluctuating between ~30,800 and 31,200 Å^2^ for the rest of the simulation time. The MT + P43 complex begins with the highest SASA (~33,200 Å^2^) and steadily decreases to ~31,000 Å^2^ by 100 ns, suggesting progressive surface adaptation and ligand accommodation.

**Figure 19:**
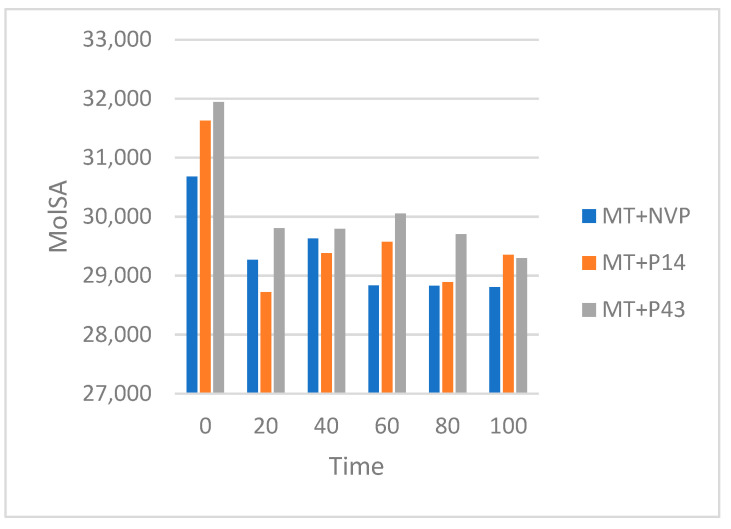
Molecular surface area (MolSA) of MT-RT complexes over a 100 ns MD simulation.

The MolSA results, presented in Table 14 and visualized in Figure 19, follow similar trends. The MT + NVP complex decreases from ~30,700 Å^2^ to ~28,900 Å^2^ by the end of the simulation, reflecting increased compactness. MT + P14 shows a sharper initial decline from ~31,700 Å^2^ to ~28,800 Å^2^ at 20 ns, followed by stabilization between ~29,200 and 29,500 Å^2^. The MT + P43 complex begins at ~32,000 Å^2^ and progressively contracts to ~29,200 Å^2^ by 100 ns, indicating enhanced molecular packing and ligand-induced structural tightening.

## 3. Discussion

### 3.1. Molecular Alignment

The molecular alignment process plays a crucial role in determining the accuracy and reliability of 3D-QSAR models. By aligning the dataset using SYBYL-X 2.0, we ensured that spatial and electronic features were uniformly represented across all compounds to maintain consistency in the analysis. The use of energy minimization with the Tripos force field and Gasteiger–Hückel charges further enhanced the precision of molecular conformations, providing a solid basis for the computational process.

Selecting compound **12**, the most active molecule, as the alignment template helped standardize the orientation of all structures relative to a biologically relevant conformation. This alignment strategy, illustrated in Figure 1, was essential for capturing meaningful structure–activity relationships and minimizing noise introduced by structural variability. Therefore, the alignment step significantly contributed to the robustness and predictive power of the resulting 3D-QSAR models.

### 3.2. Interpretation of QSAR Model Performance

The statistical parameters of both CoMFA and CoMSIA models demonstrate their reliability and predictive potential. The CoMFA model, with its high R^2^ (0.979), acceptable Q^2^ (0.643), and strong external validation (R^2^_test_ = 0.747), indicates a robust correlation between structural features and biological activity [26]. The high contribution of steric fields suggests that molecular bulk plays a dominant role in activity modulation.

Similarly, the CoMSIA model demonstrates strong internal consistency (R^2^ = 0.92; Q^2^ = 0.546) [26] and provides additional insights by incorporating donor field contributions, which account for 22.6% of the model’s explanatory power. These results suggest that donor interactions, in conjunction with steric and electrostatic contributions, play a significant role in modulating the biological activity of the compound series.

The residual analysis illustrated in Table 2, showing predicted vs. experimental values mostly within ±0.2, further supports the predictive strength of both models. These findings confirm that the models are suitable for guiding the design of new analogs with improved bioactivity.

### 3.3. Insights from CoMFA/CoMSIA Contour Maps and Their Implications for the Drug Design

The contour maps generated by CoMFA and CoMSIA models provided valuable spatial guidance for structure optimization. In both models, we found that steric and electrostatic interactions are critical determinants of biological activity. The CoMFA model emphasized the importance of bulky groups in green-contoured regions and the positive effect of electronegative groups in blue-contoured regions.

The CoMSIA analysis reinforced these findings by incorporating the hydrogen bond donor field, an additional dimension not captured by CoMFA. The large cyan contours in CoMSIA maps identified specific areas where donor groups substantially enhance inhibitory activity, offering further refinement in lead optimization.

Utilizing these insights, we designed 35 new naphthyl-diarylpyrimidine derivatives, with modifications guided by favorable contour regions. As displayed in Table 3, the predicted pIC_50_ values surpass that of compound 12, indicating that these molecules may serve as promising candidates for next-generation non-nucleoside reverse transcriptase inhibitors (NNRTIs).

### 3.4. Interpretation of ADMET Profiles

#### 3.4.1. Absorption Parameters

Adequate solubility is essential for oral bioavailability, as it enables drug dissolution in gastrointestinal fluids and facilitates absorption. The moderate solubility values (−2.89 to −3.22 Log mol/L) observed across all compounds are within an acceptable range for therapeutic application [12,13,14].

This degree of solubility supports absorption through the gastrointestinal tract, helps maintain effective plasma concentrations, and aligns with pharmacological expectations for NNRTIs. Drugs such as rilpivirine have been engineered to enhance solubility, illustrating the clinical importance of this parameter in improving dosing efficiency and therapeutic outcomes [12,13].

Intestinal absorption, a key determinant of systemic exposure, is considered favorable when values exceed 30% [16]. The predictive model used in this study estimated that all compounds, except P121, meet this criterion, indicating high absorption potential through the human small intestine [15,17,18].

This supports the viability of these compounds as orally administered HIV-1 inhibitors, except P121, which may require further structural refinement to enhance its absorption.

#### 3.4.2. Distribution Parameters

In the context of HIV-1 treatment, particularly for addressing neurocognitive complications, the ability of antiretroviral agents to penetrate the central nervous system is essential. While the blood–brain barrier (BBB) permeability (logBB) provides a general measure of brain access, the log PS value offers a more precise assessment of CNS permeability, especially when evaluating non-nucleoside reverse transcriptase inhibitors (NNRTIs) [27].

For effective CNS delivery, log PS values are ideally between −5 and −4. All designed compounds, except P124, met this criterion, indicating sufficient ability to cross the BBB and reach CNS compartments [19]. This property is critical for enhancing the therapeutic effectiveness of NNRTIs, especially in managing HIV-1-related neurocognitive disorders. Drugs such as rilpivirine and efavirenz exemplify NNRTIs with strong CNS activity, underlining the importance of permeability in clinical outcomes [20].

The findings indicate that the designed molecules, excluding P124, possess adequate distribution profiles for CNS targeting, reinforcing their potential as viable therapeutic candidates for HIV-1 with CNS involvement.

#### 3.4.3. Enzymatic Metabolism

Cytochrome P450 enzymes play a central role in drug metabolism, mediating oxidative reactions that facilitate the clearance of xenobiotics from the body [21,22].

Among these, the isoforms 1A2, 3A4, 2C9, 2C19, and 2D6 account for the metabolism of approximately 90% of clinically used drugs [23]. Inhibiting these enzymes can lead to elevated plasma concentrations and increase the risk of toxicity or adverse drug–drug interactions. The absence of inhibitory effects across all tested isoforms indicates that the designed compounds are unlikely to interfere with hepatic enzyme activity. Moreover, the identification of P14, P43, 118, P120, and P124 as CYP3A4 substrates suggests that these compounds will undergo normal metabolic processing. This favorable enzymatic profile supports the metabolic compatibility of the compounds and their suitability for further development [24].

#### 3.4.4. Excretion and Total Clearance

Total clearance is a key pharmacokinetic parameter that reflects the efficiency with which a drug is removed from systemic circulation. It defines the relationship between the drug concentration in the body and its rate of elimination [16]. The observed low clearance values (<0.5 mL/min/kg) for all designed compounds, except for compound 124, with a near-zero clearance rate (0.02 mL/min/kg), suggest a slower elimination process, which could result in longer half-lives and sustained plasma concentrations. This pharmacokinetic property may be advantageous in reducing dosing frequency and improving patient adherence, particularly in the context of chronic treatments such as HIV-1 therapy.

#### 3.4.5. Toxicity Assessment

Toxicity prediction is a fundamental step in the early stages of drug development to identify compounds with acceptable safety profiles. The use of AMES and hepatotoxicity tests revealed that none of the designed molecules are predicted to exhibit mutagenic or hepatotoxic effects, supporting their suitability as potential drug candidates [28].

### 3.5. Drug-likeness Evaluation

Incorporating drug-likeness profiling is critical to ensuring that promising computational leads also meet fundamental criteria for pharmacokinetic performance. In this context, we assessed the structural attributes of our proposed inhibitors based on physicochemical descriptors aligned with the Lipinski Rule of Five and related filters, highlighted in Table 5.

Although the molecular weights of all compounds exceed the conventional 500 g/mol threshold, it is well-established that many approved antiviral agents, especially non-nucleoside reverse transcriptase inhibitors (NNRTIs), deviate from this rule due to the need for increased molecular complexity to achieve selectivity and affinity for larger binding pockets such as those in HIV-1 reverse transcriptase [29].

For example, P14 (587.58 g/mol) and P43 (586.60 g/mol) remain within a pragmatic range and compare favorably with existing NNRTIs like rilpivirine and etravirine.

The log P values of the compounds ranging from 1.53 to 4.07 reflect a favorable balance between aqueous solubility and membrane permeability. Notably, P14 (1.87), P43 (1.53), and P148 (2.25) lie within the optimal range (1.5–2.5), suggesting enhanced oral absorption potential, with a reduced risk of lipophilic toxicity or poor aqueous solubility [30].

Compounds such as P124 (log P = 4.07) remain within acceptable limits but may benefit from future optimization to reduce excessive lipophilicity.

The number of rotatable bonds in all compounds ranges from 13 to 17, slightly exceeding Veber’s suggested threshold of 10. However, this flexibility can be advantageous when targeting proteins with induced-fit or allosteric binding sites, as is the case with HIV-1 RT [31]. This enhanced flexibility likely supports the ability of these compounds to adapt to dynamic conformational changes, a key factor in overcoming resistance mutations.

Regarding the hydrogen bonding capacity, the compounds exhibit eleven to fifteen hydrogen bond acceptors and six to nine hydrogen bond donors, reflecting a strong potential for forming stabilizing interactions with the RT active or non-catalytic sites [32]. Such rich interaction profiles are critical in enhancing binding specificity, complex stability, and resistance resilience.

The bioavailability score of 0.17 for all compounds may appear modest; however, it remains consistent with early-stage lead compounds and is not a disqualifying factor [33]. Many successful NNRTIs, including those approved for clinical use, required later-stage chemical optimization to enhance oral bioavailability without compromising efficacy [34].

Taken together, these findings demonstrate that despite modest deviations from traditional rules, the designed compounds, particularly P14 and P43, display well-balanced drug-likeness profiles, with favorable lipophilicity, binding-friendly hydrogen bonding potential, and a structural framework compatible with high-affinity RT inhibition. These properties, in combination with previously reported ADMET, docking, and MD simulation results, reinforce their promise as lead compounds for further development in the context of HIV-1 reverse transcriptase inhibition.

### 3.6. Relevance of Docking Results

The molecular docking study supports the potential of P14 and P43 as potent NNRTI candidates, as evidenced by their superior binding affinities compared to the reference compound NVP in both WT and MT reverse transcriptase, as displayed in Table 6. The enhanced docking scores of P14 and P43 suggest more stable ligand–protein complexes, a critical feature for effective enzyme inhibition [35]. The presence of conventional hydrogen bonds and electrostatic Pi–cation interactions, particularly with LYS101 in the MT-RT, highlights their capability to establish primary stabilizing forces, in contrast to NVP, which relies solely on weaker hydrophobic and Pi–Pi stacking interactions.

The SphereObject coordinate analysis and subsequent superimposition further validated the robustness of the docking protocol, showing close spatial alignment between docked and native ligand positions, particularly for P14 and P43, as highlighted in Table 7. This indicates that both compounds fit well within the active site geometry and are capable of maintaining their conformational orientation even in the presence of resistance-associated mutations.

Furthermore, the ability of P14 and P43 to interact with key residues underscores their adaptability to both enzyme forms, which is vital for overcoming drug resistance. The detailed interaction profiles presented in Table 8 confirm that P14 and P43 form more extensive and stronger stabilizing interactions than NVP, resulting in greater inhibition potential. These findings reinforce the viability of P14 and P43 as strong candidates for further development as next-generation NNRTIs capable of addressing both wild-type and resistant forms of HIV-1 reverse transcriptase.

### 3.7. Molecular Dynamics Simulation Insights

#### 3.7.1. Wild-Type Native Protein

The molecular dynamics (MD) simulation of the wild-type HIV-1 reverse transcriptase (WT-RT) and its complexes with nevirapine (NVP), P14, and P43 revealed critical insights into ligand-induced structural stability. The RMSD analysis showed that the WT-native protein maintained consistent conformational integrity, confirming its structural robustness in the absence of ligand binding. Notably, binding with P43 resulted in the lowest RMSD profile (~0.5–1.0 nm), even slightly more stable than the WT-NVP complex (~0.6–1.2 nm), indicating that P43 contributes positively to global conformational stability. In contrast, P14 binding led to a broader RMSD range (~0.6–1.4 nm), suggesting more pronounced structural deviations over time.

The RMSF analysis further supported these findings, showing that while most residue fluctuations were comparable across systems (~0.2–0.8 nm), WT + P14 exhibited elevated flexibility in loop regions, an observation that correlates with its higher RMSD. These local instabilities may reflect suboptimal engagement with dynamic protein regions or less effective stabilization of conformational hotspots.

The radius of gyration (Rg) profiles reinforced the interpretation of structural compaction. WT + P43 presented the most compact conformation (~3.0–3.3 nm), followed by WT + NVP (~3.1–3.4 nm). The WT + P14 complex displayed the highest Rg values (~3.3–3.7 nm), indicating a more expanded conformation, likely due to increased flexibility or reduced packing efficiency.

Together, these findings suggest that P43 preserves native-like dynamics, exhibiting a stabilizing effect comparable to nevirapine across all evaluated parameters. As shown in Table 9, the WT + P43 complex maintained lower RMSD and Rg values and exhibited limited residue fluctuations, indicating strong structural stability. In contrast, P14, despite its favorable binding affinity observed in docking studies, resulted in higher RMSD and a more expanded conformation. These characteristics suggest that P14 may require further structural optimization to reduce conformational perturbation and improve its stability within the WT-RT binding pocket.

#### 3.7.2. Mutant-Type Native Protein

The molecular dynamics simulations of the mutant-type reverse transcriptase (MT-RT) and its ligand-bound complexes revealed clear differences in structural stability and flexibility. The MT-native protein showed the highest level of instability, with RMSD values reaching up to 1.5 nm, RMSF peaks of approximately 1.2 nm, and moderate radius of gyration (Rg) values (~3.2–3.3 nm). These findings indicate that, in the absence of a ligand, the mutant protein is naturally more flexible and less stable.

Ligand binding significantly improved the structural stability of MT-RT, especially with nevirapine (NVP). The MT + NVP complex exhibited the lowest RMSD values (~0.4–0.7 nm), minimal residue-level fluctuations, and compact Rg values (~3.1–3.3 nm), suggesting that NVP provides strong structural stabilization.

The MT + P14 complex also stabilized the protein, although less effectively than NVP. It showed moderate RMSD values (~0.5–1.0 nm), reduced residue mobility, and a slightly more expanded structure (~3.4–3.6 nm). In comparison, the MT + P43 complex displayed the most noticeable structural expansion (~3.5–3.7 nm), along with relatively higher RMSD and RMSF values. These trends indicate that P43 contributes to increased flexibility and reduced compactness.

Overall, the results indicate that nevirapine provides the greatest conformational stability to the mutant-type reverse transcriptase (MT-RT), followed by P14, which offers moderate stabilization. As shown in Table 10, the MT + NVP complex maintained the lowest RMSD and RMSF values, along with a compact radius of gyration, highlighting its stabilizing effect on the mutant protein. In contrast, P43—while demonstrating strong binding affinity in earlier docking studies—displayed the highest Rg values and greater residue flexibility, suggesting increased structural expansion and reduced compactness. These findings reinforce the need for the further refinement of P43 to improve its dynamic performance and enhance its compatibility with the MT-RT binding pocket.

#### 3.7.3. Comparative Summary of Molecular Dynamics Simulation Across WT-RT and MT-RT Complexes

The comparative molecular dynamics simulations of wild-type (WT-RT) and mutant-type (MT-RT) HIV-1 reverse transcriptase complexes revealed clear distinctions in ligand-induced stabilization and structural dynamics. While nevirapine (NVP) consistently demonstrated strong stabilizing effects across both systems, the behavior of P14 and P43 varied, depending on the RT variant.

In the WT-RT system, P43 exhibited the most favorable stability profile, maintaining highly consistent structural dynamics and achieving the most compact conformation among the tested ligands. Notably, P43 outperformed even nevirapine, preserving native-like behavior and indicating its strong potential as a stabilizing agent. NVP also stabilized the WT-RT complex effectively, though to a slightly lesser extent. By comparison, the WT + P14 complex displayed increased conformational fluctuations and a more expanded structure, suggesting that further optimization may be needed to improve its compatibility with the WT-RT binding environment.

In the MT-RT system, the unbound mutant protein was the least stable, showing high conformational variability. Among the ligand-bound systems, NVP again provided the greatest stabilization, promoting structural compactness and suppressing local flexibility. P14 offered moderate stabilizing effects, while P43 exhibited more variable dynamics, characterized by increased flexibility and reduced compactness relative to its performance in the WT-RT system.

As summarized in Table 9 and Table 10, these findings confirm P43’s superior stabilizing capacity in the WT-RT context, while highlighting its reduced dynamic performance in the mutant form. In contrast, P14 exhibits stronger stabilization in the MT-RT system, with comparatively reduced performance in the wild-type complex. This complementary behavior suggests distinct structural compatibilities, underscoring the potential of each compound for selective optimization against specific RT variants.

### 3.8. Surface Area Analysis: SASA and MolSA

We computed the solvent accessible surface area (SASA) and molecular surface area (MolSA) to further explore the RT–ligand complexes’ structural behavior and surface exposure during the 100 ns MD simulations. These parameters provide insight into each system’s compactness and solvent interaction profile.

#### 3.8.1. Wild-Type Native Protein

The surface area analyses of wild-type reverse transcriptase (WT-RT) complexes offer important insights into the dynamic behavior of each ligand-bound system. The SASA and MolSA data collectively illustrate how each ligand influences the exposure and compactness of the protein structure over time.

The WT + NVP complex maintained relatively stable SASA values, indicating that nevirapine does not significantly disrupt the native conformation of the RT protein and preserves consistent solvent accessibility throughout the simulation. The gradual reduction in MolSA further supports the view that the protein undergoes progressive but controlled compaction, achieving a stable and well-packed conformation after the initial equilibration phase.

The WT + P14 complex showed similar behavior to NVP. Despite a slightly higher initial SASA, the fluctuations remained within a narrow range, suggesting that P14 does not induce significant structural disturbance. Its MolSA trend mirrored that of NVP, showing a gradual and consistent decline, indicating stable molecular packing. This reflects the favorable accommodation of P14 within the binding pocket, supporting its role as a stable ligand.

The WT + P43 complex, however, displayed the most pronounced reductions in both SASA and MolSA. The steady decrease in the solvent accessible surface area and marked decline in MolSA point to a notable compaction of the protein structure. This suggests that P43 promotes a tighter conformational fit, possibly inducing closure or deeper embedding within the binding cavity. While this enhanced compactness may be beneficial for ligand retention, it may also affect the protein’s flexibility and dynamics.

Overall, these results displayed in Table 11 and Table 12 demonstrate that both NVP and P14 maintain the structural integrity and solvent exposure of the WT-RT protein, while P43 drives a stronger conformational tightening. This may indicate higher binding strength, though further evaluation is needed to assess the functional implications of this reduced surface accessibility.

#### 3.8.2. Mutant-Type Native Protein

The SASA and MolSA analyses of mutant-type reverse transcriptase (MT-RT) complexes highlight how each ligand influences structural flexibility, compactness, and adaptation in a resistance-prone context. These results are particularly valuable for understanding how the ligands behave under altered structural conditions.

The MT + NVP complex showed a gradual and consistent reduction in SASA and MolSA values, indicating progressive structural tightening and stabilization. This suggests that nevirapine maintains its stabilizing effect even in the presence of resistance-associated mutations, promoting compaction and reduced solvent exposure.

The MT + P14 complex demonstrated a sharp initial decrease in both SASA and MolSA, followed by stabilization over the remainder of the simulation. This rapid early contraction may reflect an immediate conformational adjustment upon ligand binding, followed by structural accommodation and retention. The relatively stable surface profiles after 20 ns suggest that P14 forms a stable complex with MT-RT, maintaining moderate surface accessibility and compactness.

The MT + P43 complex began with the highest SASA and MolSA values, indicating initial structural looseness. However, both values steadily declined throughout the simulation, suggesting a gradual but continuous adaptation of the protein to the ligand. The final values suggest that P43 induces significant molecular packing and structural tightening, although at a slower pace compared to P14. This behavior may reflect a more progressive binding process or delayed conformational adjustment.

As summarized in Table 13 and Table 14, NVP continues to demonstrate strong stabilizing behavior in the mutant RT form. P14 shows a triggered stabilizing effect followed by steady retention, while P43 gradually promotes structural compaction. These patterns support the potential of P14 and P43 as viable candidates, with P14 showing more immediate adaptability and P43 exhibiting a slower but consistent structural tightening effect.

#### 3.8.3. Comparative Summary of Surface Area Profiles Across WT-RT and MT-RT Complexes

The comparative analysis of SASA and MolSA across wild-type and mutant-type RT complexes reveals distinct trends in ligand-induced surface behavior and structural adaptation. In the WT-RT system, nevirapine and P14 both maintained stable solvent accessibility and gradual surface compaction, indicating effective and balanced ligand accommodation. P43, in contrast, induced the strongest conformational tightening, as shown by the most substantial reductions in both SASA and MolSA, suggesting enhanced molecular packing but potentially reduced flexibility. In the MT-RT system, all three ligands promoted surface contraction to varying degrees. Nevirapine again demonstrated progressive stabilization, while P14 exhibited a sharp initial reduction followed by stability, reflecting quick adaptation. P43 showed a more gradual, sustained decrease in both SASA and MolSA, pointing to a slower, but effective, conformational adjustment.

Overall, these trends suggest that P14 exhibits a consistent and prompt stabilizing effect across both RT variants, while P43 drives a more pronounced, yet delayed, structural tightening, especially in the mutant context. Nevirapine remains the reference standard for stability, showing reliable surface behavior in both forms.

### 3.9. Overall Structural Dynamics and Surface Behavior Correlation

The combined interpretation of molecular dynamics simulation (RMSD, RMSF, Rg) and surface area analysis (SASA and MolSA) provides a consistent and well-aligned understanding of the structural behavior of HIV-1 reverse transcriptase in both wild-type (WT-RT) and mutant-type (MT-RT) forms. The results from both approaches support each other and confirm the ligand-dependent differences in conformational stability, compactness, and adaptability.

In the WT-RT system, molecular dynamics data showed that P43 provides the strongest stabilizing effect, displaying the lowest conformational fluctuations and the most compact structure. This observation is fully supported by SASA and MolSA analyses, where P43 induced the greatest reduction in surface area, indicating enhanced packing and protein–ligand closure. Nevirapine also maintained high stability and compactness across both evaluations, confirming its role as a reliable reference inhibitor. P14, by contrast, showed greater flexibility and a more expanded structure, as reflected in both the MD and surface data, suggesting that further refinement is needed to improve its compatibility with the wild-type protein.

In the MT-RT system, both methods confirmed that nevirapine again stabilizes the protein most effectively. P14 showed a rapid initial compaction and maintained moderate structural stability throughout the simulation, consistent across both RMSD/RMSF and SASA/MolSA results. Interestingly, P43 showed a slower, gradual compaction and relatively higher structural variability, indicating that it is less effective at stabilizing the mutant form compared to its strong performance in the wild-type system.

Together, these findings demonstrate strong agreement between the molecular dynamics and surface area evaluations. Both methods highlight the complementary strengths of P14 and P43: P43 is more favorable for stabilizing the wild-type RT, while P14 performs better with the mutant-type RT. This structural complementarity suggests that both molecules have value for further development, with optimization efforts potentially tailored to their respective target forms.

The comparative overview of molecular dynamics and surface behavior findings for each ligand across WT-RT and MT-RT systems is provided in Table 15, highlighting the consistency between structural dynamics and surface compaction trends.

## 4. Materials and Methods

### 4.1. Experimental Databases

In the current study, a set of 33 novel naphthyl-diarylpyrimidines derivatives were used as potent non-nucleoside reverse transcriptase inhibitors (NNRTIs); these compounds were synthesized by Xin Jin et al. in 2021 [36]. The novel naphthyl-diarylpyrimidines derivatives’ inhibitory activities are referred to by IC_50_ values (concentration required for 50% inhibition of Reverse Transcriptase activity), and the current values are converted to pIC_50_ (−log IC_50_) and employed in 3D-QSAR analysis as a dependent variable.

### 4.2. Molecular Minimization and Alignment

Energy minimization is a crucial step in generating 3D-QSAR models. Therefore, we designed the structures of the 33 compounds and optimized them using SYBYL-X 2.0 software. In the next step, we performed the molecular alignment of the dataset using the most active compound, number **12**, to align the other compounds.

### 4.3. Elaboration of 3D-QSAR Models

CoMSIA (Comparative Molecular Similarity Indices Analysis) and CoMFA (Comparative Molecular Field Analysis) are the most common techniques used in SYBYL-X 2.0 (Certara USA, Inc., Princeton, NJ, USA) to predict and analyze the relationship between the three-dimensional structures of the compounds and their biological activities. In the CoMFA study, steric and electrostatic descriptors are determined, while in the CoMSIA study, more descriptors are incorporated, notably electrostatic, hydrophobic, hydrogen bond donor, and hydrogen bond acceptor descriptors.

### 4.4. Partial Least Squares (PLS) Analysis

We incorporated partial least squares (PLS) analysis in the generation and validation of 3D-QSAR models. We performed this analysis in two steps; the Leave-One-Out method is applied to determine the optimal number of components (N) and the cross-validation coefficient (Q^2^), and the non-cross-validation method is applied using the optimal number of components (N) determined previously to compute the coefficient of determination (R^2^), the standard estimation error (SEE), and the value of the Fisher test (F). Furthermore, the best CoMFA and CoMSIA models are selected based on the highest values of Q^2^ and R^2^ (Q^2^ > 0.5 [37,38] and R^2^ > 0.6 [37,38,39]).

An optimal number of components and the lowest value of the standard estimation error (SEE) were used. Moreover, external validation is also required to investigate the reliability and the predictive ability of the generated model; therefore, a test set of five molecules was employed to determine the value of R^2^_test_, which should be greater than 0.6 [39]. Mainly, the predictive performance and statistical reliability of the model are evaluated using key statistical parameters, including the cross-validation coefficient (Q^2^), the coefficient of determination (R^2^), and the standard error of estimation (SEE).

### 4.5. ADMET and Drug-likeness Prediction

ADMET prediction is a computational technique used to evaluate novel drug candidates [40] by the estimation of their physicochemical and pharmaceutical parameters, including the adsorption, distribution, metabolism, excretion, and toxicity [41,42].

For the in silico calculations of the designed drugs’ properties, the online tool pkCSM [43] was chosen to predict the ADMET parameters.

We evaluated drug-likeness properties to determine the suitability of the designed compounds for oral administration. We computed key physicochemical properties, including the molecular weight, Log P, number of rotatable bonds, hydrogen bond donors and acceptors, and bioavailability score using the SwissADME (Modelling Group, University of Lausanne and SIB Swiss Institute of Bioinformatics, Lausanne, Switzerland platform. We assessed these values against standard drug-likeness rules such as Lipinski’s Rule of Five to support compound selection and guide further optimization.

### 4.6. Molecular Docking Analysis

We performed molecular docking studies, using AutoDockTools (ADT) and MGLTools (Center for Computational Structural Biology, The Scripps Research Institute, La Jolla, CA, USA) to investigate the orientation of a drug candidate (ligand) into the active site of a macromolecule (protein); therefore, molecular docking plays a major role in the discovery and the design of new drug candidates, based on the types of interactions between the protein and the ligand.

### 4.7. Molecular Dynamics Simulations

The molecular dynamics simulation technique, it was performed using **GROMACS** (Royal Institute of Technology, Stockholm, Sweden; Uppsala University, Uppsala, Sweden), it illustrates the temporal evolution of a biological system over time under specified thermodynamic parameters (temperature, pressure, and volume) [44,45].

It gives highly realistic models because it considers water molecules and all the degrees of freedom in the biological system, including the ligand, protein, and solvent. However, despite requiring more computational time than the previously discussed methods, the molecular dynamics (MD) simulation approach is more effective for studying complex biomolecular systems [46].

### 4.8. SASA and MolSA Analysis

To investigate the surface behavior and compactness of the protein–ligand complexes, the solvent accessible surface area (SASA) and molecular surface area (MolSA) analyses were performed throughout the 100 ns molecular dynamics simulations using BIOVIA Discovery Studio 2024 Client (Dassault Systèmes BIOVIA, San Diego, CA, USA). The SASA was calculated to estimate the total surface area of each protein–ligand complex that is accessible to solvent molecules, providing insights into conformational changes and structural exposure over time. The MolSA was used to evaluate the overall compactness of the complexes and to track how the structural arrangement evolved during the simulation. Snapshots were extracted at regular intervals (every 20 ns) from the simulation trajectories of both wild-type and mutant-type reverse transcriptase complexes. These values were then plotted and compared across different ligand-bound systems to assess the influence of each compound on the structural organization and stability of the protein.

## 5. Conclusions

This study employed a comprehensive computational strategy to design and evaluate novel non-nucleoside reverse transcriptase inhibitors (NNRTIs) against HIV-1. Using 3D-QSAR modeling, ADMET profiling, molecular docking, and molecular dynamics simulations, we identified two promising candidates, P14 and P43. The molecular dynamics analysis revealed that P43 exhibited the greatest structural stability and compactness within the wild-type reverse transcriptase (WT-RT), outperforming nevirapine in both global dynamics and molecular packing. In contrast, P14 demonstrated superior stabilization in the mutant RT (MT-RT), with consistent structural adaptation and reduced flexibility.

These findings were further supported by SASA and MolSA analyses, which confirmed ligand-induced compaction trends and solvent exposure. P43 showed the strongest decrease in the SASA and MolSA in WT-RT, indicating enhanced molecular packing and tight binding. Meanwhile, P14 maintained stable surface profiles in both RT forms, suggesting a balanced interaction and adaptability.

Both compounds also met drug-likeness criteria, with favorable binding affinities, acceptable bioavailability scores, and no predicted toxicity. Collectively, the results suggest that P43 is best suited for stabilizing WT-RT, while P14 offers greater compatibility with MT-RT, making them complementary candidates for further structural optimization and experimental validation as potential HIV-1 NNRTIs.

## Figures and Tables

**Table 1:** Statistical parameters of PLS analysis for CoMFA and CoMSIA models.

Model	Q^2^	R^2^	SEE	N	R^2^_test_	F	Fractions (%)				
							Steric	Electrostatic	Donor	Acceptor	Hydrophobic
CoMFA	0.643	0.979	0.067	5	0.747	200.37	63.7	36.3	-------------	------------	--------------------
CoMSIA	0.546	0.920	0.126	4	0.603	66.51	47.0	30.4	22.6	------------	--------------------

**Table 2:** Observed vs. predicted pIC_50_ values and residuals of naphthyl-diarylpyrimidine derivatives.

Compounds No	Observed pIC_50_	CoMFA		CoMSIA	
		Predicted	Residuals	Predicted	Residuals
**7**	7.70	7.70	0.00	7.68	−0.02
**9**	7.22	7.07	−0.15	7.14	−0.08
**10**	6.96	7.12	0.16	7.20	0.24
**12**	7.70	7.69	−0.01	7.41	−0.29
**13**	6.85	6.93	0.08	7.02	0.17
**15**	6.51	6.46	−0.05	6.56	0.05
**16**	6.59	6.56	−0.03	6.53	−0.06
**17**	6.22	6.33	0.11	6.39	0.17
**18**	6.40	6.36	−0.04	6.33	−0.07
**20**	6.57	6.59	0.02	6.43	−0.14
**21**	5.92	5.95	0.03	5.97	0.04
**22**	6.21	6.21	0.00	6.10	−0.11
**23**	6.49	6.47	−0.02	6.53	0.04
**24**	6.60	6.58	−0.02	6.76	0.16
**25**	6.85	6.83	−0.02	6.90	0.05
**26**	7.10	7.13	0.03	7.11	0.01
**27**	7.05	7.04	−0.01	7.00	−0.05
**28**	6.96	6.92	−0.04	7.04	0.08
**29**	6.68	6.64	−0.04	6.72	0.04
**30**	6.36	6.40	0.04	6.38	0.02
**31**	6.55	6.57	0.02	6.46	−0.09
**32**	6.52	6.48	−0.04	6.44	−0.08
**34**	6.80	6.90	0.10	6.75	−0.05
**35**	6.74	6.74	0.00	6.86	0.12
**36**	7.22	7.20	−0.02	7.01	−0.21
**37**	6.72	6.65	−0.07	6.70	−0.02
**38**	7.10	7.08	−0.02	7.12	0.02
**39**	7.00	7.01	0.01	7.05	0.05
**8** *	6.62	7.11	0.49	7.22	0.60
**11** *	6.77	7.41	0.64	7.32	0.55
**14** *	6.60	6.95	0.35	7.01	0.41
**19** *	6.29	6.71	0.42	6.49	0.20
**33** *	6.70	7.00	0.30	6.77	0.07

* Test set.

**Table 3:** Structural variants of designed NNRTIs and their predicted pIC_50_ values from CoMFA and CoMSIA 3D-QSAR models.

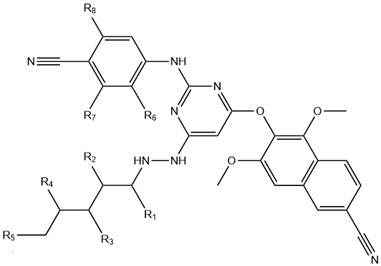										
	R1	R2	R3	R4	R5	R6	R7	R8	pIC_50pred_	
									CoMFA	CoMSIA
**P101**	C(CH_3_)_3_	H	OH	H	OH	NH_2_	NH_2_	C(CH_3_)_3_	7.745	7.832
**P103**	OH	H	C(CH_3_)_3_	H	NH_2_	OH	C(CH_3_)_3_	C(CH_3_)_3_	7.774	7.992
**P104**	C(CH_3_)_3_	H	NH_2_	C(CH_3_)_3_	OH	Cl	H	NH_2_	7.747	8.005
**P107**	C(CH_3_)_3_	H	NH_2_	C(CH_3_)_3_	OH	Cl	COR	NH_2_	7.786	7.992
**P108**	OH	H	C(CH_3_)_3_	H	NH_2_	COOH	OH	NH_2_	7.819	7.963
**P109**	C(CH_3_)_3_	H	NH_2_	C(CH_3_)_3_	OH	COR	OH	NH_2_	7.802	8.108
**P110**	OH	H	C(CH_3_)_3_	H	NH_2_	Cl	COR	NH_2_	7.777	7.863
**P111**	C(CH_3_)_3_	H	NH_2_	C(CH_3_)_3_	OH	NH_2_	OH	COOH	7.827	7.975
**P113**	C(CH_3_)_3_	H	NH_2_	C(CH_3_)_3_	OH	Cl	OH	COR	7.805	8.052
**P115**	C(CH_3_)_3_	H	NH_2_	C(CH_3_)_3_	OH	NH_2_	COR	COR	7.856	7.989
**P116**	C(CH_3_)_3_	NH_2_	NH_2_	C(CH_3_)_3_	OH	Cl	COR	NH_2_	7.782	7.932
**P118**	C(CH_3_)_3_	NH_2_	NH_2_	C(CH_3_)_3_	OH	COR	OH	NH_2_	7.795	7.954
**P120**	C(CH_3_)_3_	NH_2_	NH_2_	C(CH_3_)_3_	OH	NH_2_	OH	COOH	7.796	7.773
**P121**	OH	NH_2_	C(CH_3_)_3_	NH_2_	NH_2_	OH	COOH	NH_2_	7.741	7.709
**P122**	C(CH_3_)_3_	NH_2_	NH_2_	C(CH_3_)_3_	OH	Cl	OH	COR	7.734	7.966
**P124**	C(CH_3_)_3_	NH_2_	NH_2_	C(CH_3_)_3_	OH	NH_2_	COR	COR	7.754	8.003
**P125**	C(CH_3_)_3_	OH	NH_2_	C(CH_3_)_3_	OH	Cl	COR	NH_2_	7.805	7.998
**P127**	C(CH_3_)_3_	OH	NH_2_	C(CH_3_)_3_	OH	COR	OH	NH_2_	7.722	7.994
**P129**	C(CH_3_)_3_	OH	NH_2_	C(CH_3_)_3_	OH	NH_2_	OH	COOH	7.829	7.865
**P131**	C(CH_3_)_3_	OH	NH_2_	C(CH_3_)_3_	OH	Cl	OH	COR	7.733	8.056
**P134**	C(CH_3_)_3_	Cl	NH_2_	C(CH_3_)_3_	OH	Cl	COR	NH_2_	7.843	8.018
**P136**	C(CH_3_)_3_	Cl	NH_2_	C(CH_3_)_3_	OH	COR	OH	NH_2_	7.765	7.971
**P138**	C(CH_3_)_3_	Cl	NH_2_	C(CH_3_)_3_	OH	NH_2_	OH	COOH	7.764	7.968
**P14**	OH	OH	OH	H	OH	H	H	H	7.733	8.217
**P140**	C(CH_3_)_3_	Cl	NH_2_	C(CH_3_)_3_	OH	Cl	OH	COR	7.763	8.099
**P142**	C(CH_3_)_3_	Cl	NH_2_	C(CH_3_)_3_	OH	NH_2_	COR	COR	7.724	8.050
**P143**	C(CH_3_)_3_	COR	NH_2_	C(CH_3_)_3_	OH	Cl	COR	NH_2_	7.827	8.137
**P147**	C(CH_3_)_3_	COR	NH_2_	C(CH_3_)_3_	OH	NH_2_	OH	COOH	7.790	7.984
**P148**	OH	COR	C(CH_3_)_3_	COR	NH_2_	OH	COOH	NH_2_	7.758	7.738
**P149**	C(CH_3_)_3_	COR	NH_2_	C(CH_3_)_3_	OH	Cl	OH	COR	7.758	8.157
**P151**	C(CH_3_)_3_	COR	NH_2_	C(CH_3_)_3_	OH	NH_2_	COR	COR	7.853	7.914
**P40**	OH	OH	H	H	OH	H	H	H	7.729	7.716
**P43**	OH	OH	OH	H	NH_2_	H	H	H	7.721	8.145
**P86**	OH	OH	H	H	OH	NH_2_	H	Cl	7.710	7.957
**P90**	NH_2_	OH	H	H	H	C(CH_3_)_3_	H	H	7.724	7.823

**Table 4:** ADMET prediction results: absorption, distribution, metabolism, excretion, and toxicity profiles.

	Absorption		Distribution		Metabolism							Excretion	Toxicity	
Best Picks	Water Solubility Log mol/L	Intestinal Absorption (Human) %	BBB Permeability LogBB	CNS Permeability Log PS	CYP	Inhibitor						Total Clearance mL/min/Kg	AMES Toxicity	Hepatotoxicity
					Substrate		1A2	2C19	2C9	2D6	3A4			
					2D6	3A4								
**P14**	−3.04	46.86	−2.32	−4.38	No	Yes	No	No	No	No	No	0.47	No	No
**P40**	−3.22	70.17	−2.33	−4.16	No	No	No	No	No	No	Yes	0.48	No	No
**P43**	−2.94	60.37	−2.70	−4.67	No	Yes	No	No	No	No	No	0.45	No	No
**P86**	−3.00	66.54	−2.26	−4.28	No	No	No	No	No	No	No	0.36	No	No
**P118**	−2.89	54.94	−3.00	−4.10	No	Yes	No	No	No	No	Yes	−0.24	No	No
**P120**	−2.89	39.20	−2.95	−4.05	No	Yes	No	No	No	No	No	−0.31	No	No
**P121**	−2.89	23.23	−3.21	−4.74	No	No	No	No	No	No	No	−0.25	No	No
**P124**	−2.90	64.35	−2.73	−3.71	No	Yes	No	No	No	No	No	0.02	No	No
**P129**	−2.89	43.84	−2.98	−4.04	No	No	No	No	No	No	No	−0.18	No	No
**P148**	−2.89	30.46	−2.57	−4.15	No	No	No	No	No	No	No	−0.24	No	No

**Table 5:** Drug-likeness parameters of the designed compounds.

Compounds	Molecular Weight (g/mol)	Log P	Rotatable Bonds	H-Bond Acceptors	H-Bond Donors	Bioavailability Score
**P14**	587.58	1.87	13	12	7	0.17
**P40**	571.58	2.65	13	11	6	0.17
**P43**	586.60	1.53	13	12	7	0.17
**P86**	621.04	2.71	13	11	7	0.17
**P118**	769.89	3.23	16	14	9	0.17
**P120**	756.85	2.81	16	14	9	0.17
**P121**	712.80	2.84	15	13	8	0.17
**P124**	780.91	4.07	17	13	7	0.17
**P129**	757.84	2.73	16	14	9	0.17
**P148**	769.80	2.25	17	15	8	0.17

**Table 6:** Binding affinity values of ligands to wild-type and mutant-type reverse transcriptase (kcal/mol).

	WT	MT
**NVP**	−9.1	−10.0
**P14**	−12.5	−10.5
**P40**	−12.5	−9.2
**P43**	−13.0	−10.7
**P86**	−12.1	−10.5
**P118**	−11.1	−10.1
**P120**	−11.6	−9.2
**P129**	−12.4	−9.8
**P148**	−9.5	−9.5

**Table 7:** Coordinates of SphereObject attributes (XYZ) for ligand-bound wild-type and mutant-type reverse transcriptase complexes.

	Compounds	SphereObject Attributes (XYZ)		
		X	Y	Z
**WT**	**WT + NVP**	143.342190	−23.723619	72.781571
	**WT + P14**	140.065080	−20.230380	68.564120
	**WT + P43**	139.728078	−19.907275	68.630824
**MT**	**MT + NVP**	41.220810	52.715143	49.583619
	**MT + P14**	39.756720	45.312020	49.723920
	**MT + P43**	39.390137	44.873216	49.682137

**Table 8:** Types of interactions between ligands (P14, P43, and NVP) and reverse transcriptase receptor (WT and MT) with corresponding binding affinity values.

			Hydrogen Bonds				Electrostatic Interactions		Hydrophobic Interactions				Pi-Pi Stacking Interactions			
			Conventional Hydrogen Bonds		Carbon Hydrogen Bonds		Pi-Cation		Pi-Alkyl		Pi-Sigma		Pi-Pi Stacked		Pi-Pi T-Shaped	
Proteins	Ligands	Binding Affinities	Nbre	Amino Acids	Nbre	Amino Acids	Nbre	Amino Acids	Nbre	Amino Acids	Nbre	Amino Acids	Nbre	Amino Acids	Nbre	Amino Acids
**WT**	NVP	−9.1	0	-----------------------------	0	--------------------	0	---------	6	LYS103 LEU100 VAL106(2) LEU234(2)	1	LEU100	2	TYR188 TRP229	2	TYR188 TRP229
	P14	−12.5	2	GLY99(2)	1	GLY190	0	---------	1	LEU100	1	LEU100	0	--------	0	--------
	P43	−13	4	GLY99(2), VAL381, ILE382	1	GLY190	0	---------	1	LEU100	1	LEU100	1	TYR181	0	--------
**MT**	NVP	−10	0	-----------------------------	1	LYS101	0	---------	4	LYS103 VAL179 LEU100 PRO95	2	LEU100 VAL106	2	TYR181 TRP229	2	TYR181 TRP229
	P14	−10.5	4	GLY99, VAL381, LYS101, ILE382	0	---------------------	1	LYS101	2	VAL179 VAL106	2	LEU100	0	--------	0	--------
	P43	−10.7	3	GLY99, LYS101, ILE382	1	PRO95	1	LYS101	2	VAL179 LYS103	2	LEU100	0	--------	0	--------

**Table 9:** Structural stability parameters (RMSD, RMSF, and radius of gyration) of native wild-type reverse transcriptase and its complexes during molecular dynamics simulation.

	RMSD (nm)	RMSF (nm)	Rg (nm)
**WT-native**	0.5–1.1	0.2–0.8	3.2–3.4
**WT-NVP**	0.6–1.2	0.2–0.8	3.1–3.4
**WT-P14**	0.6–1.4	0.2–0.8	3.3–3.7
**WT-P43**	0.5–1.0	0.2–0.8	3.0–3.3

**Table 10:** Structural stability parameters (RMSD, RMSF, and radius of gyration) of native mutant-type reverse transcriptase and its complexes during molecular dynamics simulation.

	RMSD (nm)	RMSF (nm)	Rg (nm)
**MT-native**	0.5–1.5	~1.2	3.2–3.3
**MT-NVP**	0.4–0.7	<1.2	3.1–3.3
**MT-P14**	0.5–1.0	<1.2	3.4–3.6
**MT-P43**	0.5–1.2	<1.2	3.5–3.7

**Table 11:** Solvent accessible surface area (SASA) values (Å^2^) for WT-RT complexes during 100 ns of MD simulation.

Time (ns)	WT + NVP	WT + P14	WT + P43
0	31,700	32,200	31,800
20	31,500	31,900	31,100
40	32,000	31,800	31,000
60	31,700	31,600	30,400
80	31,600	31,800	30,800
100	31,500	31,400	30,300

**Table 12:** Molecular surface area (MolSA) values (Å^2^) for WT-RT complexes during 100 of MD simulation.

Time (ns)	WT + NVP	WT + P14	WT + P43
0	31,300	32,100	31,900
20	28,900	30,600	30,700
40	30,000	39,900	30,100
60	29,700	29,700	29,600
80	29,600	30,000	29,500
100	29,700	30,100	28,800

**Table 13:** Solvent accessible surface area (SASA) values (Å^2^) for MT-RT complexes during 100 ns MD simulation.

Time (ns)	MT + NVP	MT + P14	MT + P43
0	32,200	32,800	33,200
20	30,700	30,200	30,900
40	30,800	30,800	30,800
60	30,600	31,200	31,500
80	30,600	30,800	31,000
100	29,800	31,100	31,000

**Table 14:** Molecular surface area (MolSA) values (Å^2^) for MT-RT complexes during 100 ns MD simulation.

Time (ns)	MT + NVP	MT + P14	MT + P43
0	30,700	31,700	32,000
20	29,200	28,800	29,800
40	29,600	29,300	29,800
60	28,900	29,500	30,000
80	28,900	28,900	29,700
100	28,900	29,300	29,200

**Table 15:** Correlation of molecular dynamics parameters and surface area profiles for WT-RT and MT-RT complexes with NVP, P14, and P43.

Ligand	WT-RT Stability (MD)	WT-RT Surface Behavior	MT-RT Stability (MD)	MT-RT Surface Behavior	Overall Interpretation
**NVP**	High stability	Gradual compaction	High stability	Progressive compaction	Stable in both WT and MT
**P14**	Moderate stability	Stable compaction	Highest stability	Initial drop, stable later	Moderate in both, better in MT
**P43**	Highest stability	Strongest compaction	Variable stability	Gradual compaction	Strong in WT, weaker in MT

## Data Availability

The original contributions presented in the study are included in the article, further inquiries can be directed to the corresponding author/s.
